# Flavonoid metabolites reduce tumor necrosis factor‐α secretion to a greater extent than their precursor compounds in human THP‐1 monocytes

**DOI:** 10.1002/mnfr.201400799

**Published:** 2015-05-03

**Authors:** Jessica L. di Gesso, Jason S. Kerr, Qingzhi Zhang, Saki Raheem, Sai Krishna Yalamanchili, David O'Hagan, Colin D. Kay, Maria A. O'Connell

**Affiliations:** ^1^ School of PharmacyUniversity of East AngliaNorwichUK; ^2^ Department of NutritionNorwich Medical SchoolUniversity of East AngliaNorwichUK; ^3^ School of ChemistryUniversity of St. AndrewsSt. AndrewsUK

**Keywords:** Cytokine, Inflammation, Metabolism, Phase 2 conjugates, Polyphenol

## Abstract

**Scope:**

Flavonoids are generally studied in vitro, in isolation, and as unmetabolized precursor structures. However, in the habitual diet, multiple flavonoids are consumed together and found present in the circulation as complex mixtures of metabolites. Using a unique study design, we investigated the potential for singular or additive anti‐inflammatory effects of flavonoid metabolites relative to their precursor structures.

**Methods and results:**

Six flavonoids, 14 flavonoid metabolites, and 29 combinations of flavonoids and their metabolites (0.1–10 μM) were screened for their ability to reduce LPS‐induced tumor necrosis factor‐α (TNF‐α) secretion in THP‐1 monocytes. One micromolar peonidin‐3‐glucoside, cyanidin‐3‐glucoside, and the metabolites isovanillic acid (IVA), IVA‐glucuronide, vanillic acid‐glucuronide, protocatechuic acid‐3‐sulfate, and benzoic acid‐sulfate significantly reduced TNF‐α secretion when in isolation, while there was no effect on TNF‐α mRNA expression. Four combinations of metabolites that included 4‐hydroxybenzoic acid (4HBA) and/or protocatechuic acid also significantly reduced TNF‐α secretion to a greater extent than the precursors or metabolites alone. The effects on LPS‐induced IL‐1β and IL‐10 secretion and mRNA expression were also examined. 4HBA significantly reduced IL‐1β secretion but none of the flavonoids or metabolites significantly modified IL‐10 secretion.

**Conclusion:**

This study provides novel evidence suggesting flavonoid bioactivity results from cumulative or additive effects of circulating metabolites.

**Abbreviations**
4HBA: 4‐hydroxybenzoic acid
BA: benzoic acid
C3G: cyanidin‐3‐glucoside
IVA: isovanillic acid
NF‐κB: nuclear factor kappa‐light‐chain‐enhancer of activated B cells
P3G: peonidin‐3‐glucoside
PCA: protocatechuic acid
TACE: TNF‐α converting enzyme
TNF‐α: tumor necrosis factor‐alpha
VA: vanillic acid
VC: vehicle control

## 1 Introduction

Flavonoids are phenolic secondary plant metabolites found ubiquitously in our habitual diets 1, 2. They have been extensively studied due to the widely held hypothesis that they contribute to the positive health effects associated with fruit and vegetable consumption 3. Flavonoid consumption has been associated with a lower incidence of chronic age related diseases, including cardiovascular disease, cancer, and neurodegenerative disorders 3, 4, 5, where chronic low grade inflammation is a central underlying predisposing risk factor 6. Tumor necrosis factor‐alpha (TNF‐α) and IL‐1β are central mediators of the inflammatory process and pharmacological inhibition of these pro‐inflammatory cytokines results in improved cardiovascular health and a lower incidence of myocardial infarction 7, 8. Flavonoids have been shown to reduce TNF‐α and IL‐1β levels in human and animal studies and cell culture models 9, 10, 11. In addition, IL‐10 plays an important anti‐inflammatory role in the resolution of inflammation and has been shown to be upregulated by several flavonoids including hesperidin, quercetin, and catechin 12, 13. TNF‐α, IL‐1β, and IL‐10 are all regulated by the pro‐inflammatory transcription factor nuclear factor kappa‐light‐chain‐enhancer of activated B cells (NF‐κB), and NF‐κB has also been shown to be a target of several flavonoids including quercetin 14 and cyanidin‐3‐glucoside (C3G) 15.

**Figure 1:**
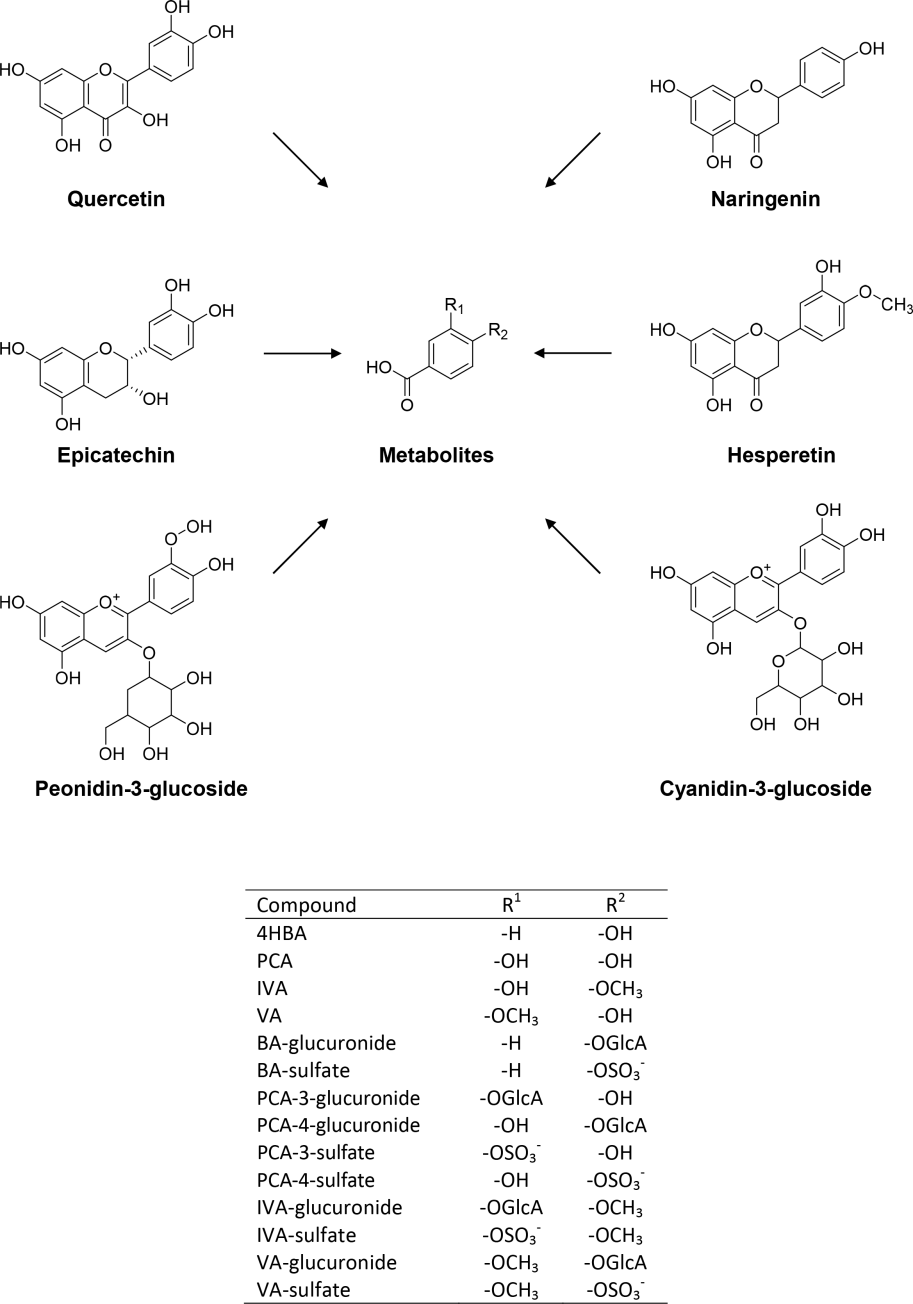
Structures of the six precursor flavonoids and their metabolites. Details of constituent groups given in inset table. –OGlc represents an oxygen‐linked glucose; –OGlcA represents an oxygen‐linked glucuronide.

Most in vitro studies conducted to date have focused on the effect of individual precursor flavonoids on inflammatory mediators 16. However, flavonoids are extensively metabolized and found in the systemic circulation as phase II conjugates or products of bacterial catabolism 17, 18. It is therefore more relevant to study the effects of flavonoid metabolites (Fig. 1) over their precursor unmetabolized structures 19. Similarly, previous studies have focused on supraphysiological concentrations of flavonoids in in vitro studies using doses often between 25 and 100 μM 16. Therefore, it is equally important to consider physiologically appropriate concentrations of flavonoid metabolites, as most are unlikely to reach levels in excess of 10 μM in the human systemic circulation 20. Lastly, in the habitual diet, flavonoids are consumed as complex mixtures of compounds, which are consequently present in the systemic circulation as diverse mixtures of precursor and metabolic conjugates. It is therefore important to consider the potential for additive, synergistic, or inhibitory effects of multiple compounds collectively. Here, we have designed a study to address these previous conceptual inaccuracies and attempted to explore the activities of flavonoid metabolites at physiologically relevant concentrations and in complex mixtures as they may be found in vivo.

In the present study, human THP‐1 monocytic cells were utilized (as previously described 21) as an in vitro screening model of inflammation, to explore the activity of flavonoid metabolites on LPS‐induced TNF‐α, IL‐1β, and IL‐10 expression. Here, six common dietary flavonoids (quercetin, naringenin, epicatechin, hesperetin, peonidin‐3‐glucoside (P3G), C3G), 14 metabolites (including synthesized methyl, glucuronide, and sulfate conjugates), and 29 unique combinations of flavonoid and metabolite were investigated. The treatments were explored across concentrations ranging from 0.1 to 10 μM (Table 1) in order to investigate the effects of more physiologically relevant concentrations of flavonoids and their metabolites 17, 19, 22, 23, as would be potentially found in the circulation following the consumption of a flavonoid‐rich diet.

**Table 1:** Constituent components of the 29 treatment combinations^a^

	Precursor flavonoids						Flavonoid metabolites												
Combination	Naringenin	C3G	Quercetin	Epicatechin	P3G	Hesperetin	PCA	VA	4HBA	BA‐glucuronide	BA‐sulfate	PCA‐3‐glucuronide	PCA‐4‐glucuronide	PCA‐3‐sulfate	PCA‐4‐sulfate	VA‐glucuronide	VA‐sulfate	IVA‐glucuronide	IVA‐sulfate
1					+	+													
2		+	+																
3		+	+	+															
4	+	+	+	+															
5		+	+	+	+														
6		+	+	+	+	+													
7	+	+	+	+	+	+													
8							+	+											
9							+		+										
10							+	+	+										
11									+	+									
12									+		+								
13									+	+	+								
14								+	+										
15							+	+	+	+	+	+	+	+	+	+	+	+	+
16							+					+							
17							+						+						
18							+					+	+						
19							+							+					
20							+								+				
21							+							+	+				
22							+					+	+	+	+				
23								+								+			
24								+									+		
25								+								+	+		
26								+										+	
27								+											+
28								+										+	+
29								+								+	+	+	+

a The concentrations used represent cumulative concentrations, e.g. a 10 μM dose consisting three analytes contains 3.33 μM of each analyte.

## 2 Materials and methods

### 2.1 Chemicals

The flavonoids hesperetin, naringenin, quercetin, epicatechin, C3G, and metabolites protocatechuic acid (PCA), isovanillic acid (IVA), vanillic acid (VA), and 4‐hydroxybenzoic acid (4HBA) were obtained from Sigma Aldrich (Poole, UK). P3G was obtained from Polyphenols Laboratories AS (Sandes, Norway). The remaining metabolites (benzoic acid (BA)‐glucuronide, BA‐sulfate, PCA‐3‐glucuronide, PCA‐4‐glucuronide, PCA‐3‐sulfate, PCA‐4‐sulfate, IVA‐glucuronide, IVA‐sulfate, VA‐glucuronide, and VA‐sulfate) were not commercially available and synthesized for the present study, as previously described 24. Flavonoids and their metabolites were dissolved in DMSO and added to the cells at a final concentration of 0.1% DMSO. Treatments containing mixtures of flavonoids or metabolites represented cumulative concentrations of 0.1, 1, 10, or 100 μM, where each component provided an equal molar concentration. For example, a 0.1 μM treatment combination comprising three components contained 0.033 μM of each component, or 0.33 μM of each component for a 1 μM treatment, or 3.3 μM of each component for a 10 μM, and similarly 33.3 μM for the 100 μM treatment. *Escherichia coli* LPS 5 mg/mL (O111:B4) was obtained from Calbiochem (San Diego, USA). SB203580 was obtained from Tocris Bioscience (Bristol, UK). DMSO and all other reagents were from Sigma, unless otherwise indicated.

### 2.2 Cell culture

The human monocytic cell line, THP‐1, was obtained from the European Collection of Cell Cultures (Health Protection Agency, Salisbury, UK) and cultured in RPMI 1640 medium (PAA Laboratories, Pasching, Austria), supplemented with 10% fetal bovine serum (Sigma), 2 mM l‐glutamine, 100 U/mL penicillin, and 100 μg/mL streptomycin (PAA Laboratories) as previously described 25. Cells were maintained in culture at 37°C in a humidified atmosphere with 5% CO_2_. Cell density was maintained between 0.3 and 0.8 × 10^6^ cells/mL and used between passages 5 and 20.

### 2.3 Cell viability assay

Cell viability was measured using the WST‐1 assay according to the manufacturer's protocol (Roche Diagnostics, Mannheim, Germany). Briefly, 0.5 × 10^5^ cells/well were seeded in 96‐well plates. Treatments were added at final concentrations of 5, 10, 50, and 100 μM. Cells were incubated for 24 h at 37°C and 5% CO_2_. A total of 10 μL WST‐1 Cell Proliferation Reagent was added for 3 h and absorbance was measured at 450 nm.

### 2.4 Cytokine protein secretion

THP‐1 cells (0.5 × 10^6^ cells/well) were seeded in 24‐well plates. Treatments were added at final concentrations of 0.1, 1, 10, and 100 μM. Plates were incubated at 37°C, 5% CO_2_ for 30 min prior to addition of LPS. For TNF‐α production, cells were stimulated with LPS (100 pg/mL) for 3 h. Cells were stimulated with 1 μg/mL LPS for 24 h for IL‐1β and IL‐10 production assays. Cytokine concentrations in supernatants were quantified using human ELISA kits from BD Biosciences (Oxford, UK). ELISAs were carried out in accordance to the manufacturer's instructions. Briefly, 100 μL of supernatant sample or standard in duplicate was incubated for 2 h at room temperature on 96‐well plates precoated with an appropriate anti‐human monoclonal antibody. Following incubation, plates were washed in PBS/0.05% Tween‐20. Immediately following washing, 100 μL of enzyme‐linked secondary antibody was added to each well and incubated for 1 h at room temperature. Plates were washed, 100 μL of substrate solution was added to each well, and incubated for 30 min, followed by the addition of 50 μL 1M H_2_SO_4_. Absorbance was determined at 450 nm. The interassay coefficients of variation were 9.9, 7.8, and 5.7% for the TNF‐α, IL‐1β, and IL‐10 ELISAs, respectively.

### 2.5 Cytokine mRNA expression

THP‐1 cells (1 × 10^6^ cells/mL) were treated with 1 μM compounds for 30 min prior to stimulation with LPS. After incubation with 100 pg/mL LPS for 2 h (TNF‐α) or 1 μg/mL LPS for 24 h (IL‐1β), cells were centrifuged and the pellet was resuspended in TRIzol Reagent (Ambion, Carlsbad, USA) and total RNA was extracted according to the manufacturer's instructions. RNA concentration was quantified using a Nanodrop ND‐1000 spectrophotometer and concentrations were adjusted to 200 ng/μL. RNA was reverse transcribed using a TaqMan kit (Roche Diagnostics) according to the manufacturer's instructions. Reverse transcription was carried out on a PTC‐100 thermocycler (Bio‐Rad, Herts, UK) using the following conditions: 21°C for 10 min, 42°C for 15 min, 99°C for 5 min, and 4°C for 5 min. Primers were obtained from Invitrogen (Paisley, UK) with the following sequences: TNF‐α forward: 5′‐GCCCAGGCAGTCAGATCATC5‐3′, reverse: 5′‐CGGTTCAGCCACTGGAGCT‐3′; IL‐1β forward: 5′‐GGACAAGCTGAGGAAGATGC‐3′, reverse: 5′‐TCGTTA TCCCATGTGTCGAA‐3′, and GAPDH forward: 5′‐AACA GCCTCAAGATCATCAGCA‐3′, reverse: 5′‐TGCTAAGCAGTTGGTGGTGC‐3′. mRNA expression was measured by real‐time PCR using a QIAGEN Rotor‐Gene Q and SYBR Green technology. DNA was replicated for 1 cycle of 95°C for 120 s, and then 40 cycles of 95°C for 15 s and 40°C for 40 s. Each mRNA expression was normalized against GAPDH mRNA expression using the standard curve method.

### 2.6 NF‐κB luciferase reporter assay

NF‐κB pGL3 firefly luciferase and pGL4.73 Renilla luciferase plasmids and the Dual‐Glo Luciferase assay were purchased from Promega (Madison, USA). One milliliter of THP‐1 cells at 1 × 10^6^ cells/mL were seeded in 24‐well plates in RPMI 1640 medium. After 24 h, cells were transfected using Lipofectamine LTX Plus reagent and Opti‐MEM reduced serum media (Invitrogen) according to the manufacturer's protocol and as previously described 26. Cells were transfected with pGL4.73 or pGL3 for 18 h. Cells were treated with 10 μM compounds for 30 min prior to LPS stimulation (100 ng/mL) for 7 h at 37°C, 5% CO_2_. Reporter activity was measured using the Dual‐Glo luciferase assay on a BMG Labtech POLARstar OPTIMA plate reader. Each firefly reading was normalized to the corresponding Renilla reading prior to analysis.

### 2.7 Statistics

Data are presented as mean ± SD, from three independent biological replicates. Treatments were compared to 0.1% DMSO vehicle control (VC). One‐way ANOVA with post‐hoc LSD (when comparing more than two conditions), or a two‐tailed independent sample *t*‐test (when comparing two conditions) was performed using PASW statistics 18 software (Chicago, USA); *p < 0.05, **p < 0.01, ***p < 0.001.

## 3 Results

### 3.1 Effects of flavonoids and their metabolites on cell viability

Flavonoids have previously been reported to affect cell viability at high concentrations 27. To ensure that the effects of the treatments in the present study were not influenced by cell viability, THP‐1 cells were treated with 5–100 μM flavonoid for 24 h and cell viability was measured by WST‐1 assay. No flavonoid or metabolite was found to affect cell viability, individually or in combination, at 5, 10, or 50 μM (data not shown). Quercetin and naringenin were the only compounds to significantly reduce cell viability at 100 μM (16.2 ± 7.6%, *p* < 0.001 and 13.2 ± 10.7%, *p* < 0.001; respectively).

### 3.2 Flavonoid metabolites inhibit TNF‐α secretion

**Figure 2:**
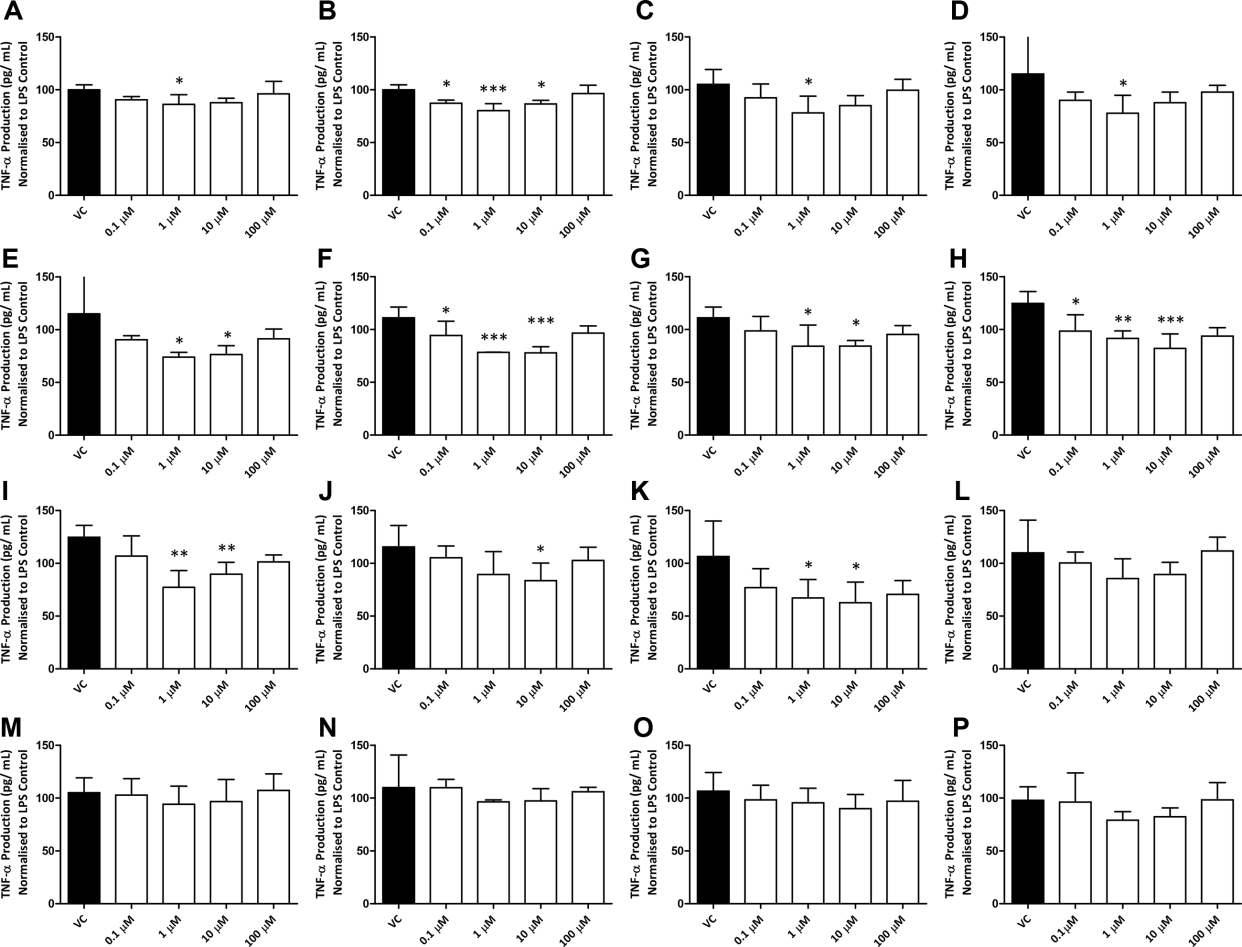
Effect of flavonoid treatments on LPS‐induced THP‐1 TNF‐α protein secretion. (A) C3G, (B) P3G, (C) IVA, (D) BA‐sulfate, (E) PCA‐3‐sulfate, (F) IVA‐glucuronide, (G) VA‐glucuronide, (H) Combination 9, (I) Combination 10, (J) Combination 13, (K) Combination 16, (L) PCA, (M) 4HBA, (N) VA, (O) BA‐glucuronide, and (P) PCA‐3‐glucuronide. Data were normalized to an LPS control and then compared to VC. Columns represent mean ± SD (*n* = 3). Each independent experiment was carried out in technical duplicate. *p < 0.05, **p < 0.01, ***p < 0.001 (one‐way ANOVA and post‐hoc LSD).

Of the 20 individual compounds and 29 combination treatments screened, two flavonoids, five metabolites, and four combinations significantly reduced LPS‐induced TNF‐α protein levels compared to VC (Table 2). Specifically, treatment with the precursor flavonoids C3G and P3G (reduction 13–20%; *p* ≤ 0.050), and the metabolites BA‐sulfate, IVA, IVA‐glucuronide, VA‐glucuronide, and PCA‐3‐sulfate (reduction 27–41%; *p* ≤ 0.040), significantly reduced LPS‐induced TNF‐α secretion. Of the combination treatments tested, treatments containing PCA and 4HBA (combination 9); PCA, VA, and 4HBA (combination 10); 4HBA, BA‐glucuronide, and BA‐sulfate (combination 13); and PCA and PCA‐3‐glucuronide (combination 16) significantly reduced TNF‐α secretion (reduction of 4–47%; *p* ≤ 0.050). The individual constituents of the active combination treatments were also assessed and did not significantly reduce TNF‐α compared to the VC at any concentration tested, up to and including 100 μM (Fig. 2).

**Table 2:** Significant effects of treatments on LPS‐induced TNF‐α protein secretion

Treatment	Average change in TNF‐α protein from VC (% ± SD)		
	0.1 μM	1 μM	10 μM
C3G	↓ 9.5 ± 2.4	↓ 13.7 ± 4.4*	↓ 12.2 ± 1.2
P3G	↓ 12.7 ± 4.2*	↓ 19.8 ± 3.1***	↓ 13.4 ± 6.5*
IVA	↓ 12.8 ± 3.3	↓ 27.0 ± 3.5*	↓ 20.2 ± 5.7
BA‐sulfate	↓ 24.9 ± 32.5	↓ 37.2 ± 40.1*	↓ 27.3 ± 38.5
PCA‐3‐sulfate	↓ 24.6 ± 33.8	↓ 41.1 ± 42.0*	↓ 38.6 ± 43.0*
IVA‐glucuronide	↓ 16.8 ± 15.1*	↓ 32.8 ± 9.8***	↓ 33.3 ± 11.8***
VA‐glucuronide	↓ 12.5 ± 15.7	↓ 26.9 ± 19.4*	↓ 26.8 ± 4.9*
Combination 9	↓ 26.3 ± 6.4*	↓ 33.1 ± 6.1**	↓ 42.7 ± 13.5***
Combination 10	↓ 17.9 ± 9.8	↓ 47.4 ± 12.3**	↓ 35.1 ± 5.1**
Combination 13	↓ 10.3 ± 29.0	↓ 26.2 ± 37.6	↓ 32.0 ± 35.4*
Combination 16	↓ 1.1 ± 19.0	↓ 6.5 ± 21.1*	↓ 4.4 ± 13.4*

Treatments (see Table 1 for details) were added to THP‐1 cells at final concentrations of 0.1, 1, and 10 μM for 30 min prior to stimulation with 100 pg/mL LPS for 3 h. LPS‐induced TNF‐α protein secretion was quantified by ELISA. Data shown are averages of three independent experiments, each carried out in technical duplicate. Analysis was performed by one‐way ANOVA and post‐hoc LSD, *p < 0.05, **p < 0.01, ***p ≤ 0.001.

### 3.3 Effects of flavonoids on LPS‐induced NF‐κB‐driven gene transcription

Flavonoids have previously been reported to inhibit NF‐κB 28, 29, which is a major regulator of TNF‐α. To determine if the flavonoids or metabolites also reduced other NF‐κB‐regulated cytokines, the bioactive treatments identified in the TNF‐α screen were investigated for effects on IL‐1β and IL‐10 secretion. Here, none of the active treatments significantly inhibited LPS‐induced IL‐1β secretion (Table 3). Interestingly, 4HBA, a component of combination 9 and 10, significantly reduced IL‐1β protein secretion (49.6 ± 12.3% versus VC, *p* = 0.011). None of the active treatments modified IL‐10 secretion (*p* ranging from 0.479 to 0.948, data not shown). These results indicate that the bioactive effects of the individual treatments and combinations are largely selective for TNF‐α and are therefore independent of NF‐κB. To verify this, an NF‐κB luciferase assay was utilized. In cells transfected with an NF‐κB reporter and stimulated with a subset of individual treatments or their combinations for 30 min prior to LPS stimulation, none of the individual treatments or combinations had any effect on LPS‐induced NF‐κB reporter activity (*p* ranging from 0.144 to 0.822, data not shown), confirming that the effects on TNF‐α are independent of NF‐κB.

**Table 3:** Effect of treatments on IL‐1β protein expression

Treatment	Average change in IL‐1β protein from vehicle control (% ± SD)
	1 μM
C3G	↓ 1.3 ± 28.9
P3G	↓ 15.2 ± 20.6
IVA	↑ 19.2 ± 10.2
IVA‐glucuronide	↑ 24.3 ± 15.3
VA‐glucuronide	↓ 1.7 ± 50.3
PCA‐3‐sulfate	↓ 31.2 ± 3.6
BA‐sulfate	↓ 28.9 ± 35.0
Combo 9	↓ 8.7 ± 17.8
Combo 10	↑ 15.5 ± 30.9
Combo 13	↓ 6.0 ± 11.7
Combo 16	↓ 10.3 ± 22.3
VA	↓ 23.0 ± 44.4
PCA	↓ 16.8 ± 15.0
4HBA	↓ 49.6 ± 12.3*
BA‐glucuronide	↓ 2.6 ± 10.5
PCA‐3‐glucuronide	↓ 2.0 ± 22.1

Treatments were added to THP‐1 cells at a final concentration of 1 μM for 30 min prior to stimulation with 10 μg/mL LPS for 24 h. Data were normalized to LPS and compared with VC. Data shown are averages of three independent experiments, each carried out in technical duplicate. Analysis was performed by two‐tailed independent samples *t*‐test, *p < 0.05.

### 3.4 Post‐transcriptional effects of flavonoids on cytokine secretion

To determine if the effects of the bioactive treatments on LPS‐induced TNF‐α were transcriptional or post‐transcriptional, cells were treated with the active treatments and TNF‐α mRNA expression was examined by RT‐qPCR. None of the bioactive treatments significantly inhibited LPS‐induced TNF‐α mRNA expression (Table 4). Indeed, one of the compounds of combination 13, BA‐glucuronide, significantly (*p* = 0.050) increased TNF‐α mRNA expression. In addition, 4HBA, which significantly inhibited LPS‐induced IL‐1β secretion (*p* = 0.001), had no effect on IL‐1β mRNA expression (*p* = 1.000).

**Table 4:** Effect of treatments on TNF‐α mRNA expression

Treatment	Average change in TNF‐α mRNA from vehicle control (% ± SD)
	1 μM
C3G	↑ 0.8 ± 13.0
P3G	↓ 7.0 ± 28.7
IVA	↓ 2.8 ± 62.7
IVA‐glucuronide	↑ 17.1 ± 51.8
VA‐glucuronide	↑ 6.3 ± 27.1
PCA‐3‐sulfate	↓ 1.3 ± 21.7
BA‐sulfate	↓ 8.8 ± 33.2
Combo 9	↑ 17.7 ± 36.0
Combo 10	↑ 9.5 ± 28.7
Combo 13	↓ 35.9 ± 65.8
Combo 16	↑ 3.6 ± 56.1
VA	↑ 20.2 ± 55.5
PCA	↑ 17.5 ± 36.0
4HBA	↑ 44.5 ± 64.6
BA‐glucuronide	↑ 23.8 ± 6.5*
PCA‐3‐glucuronide	↑ 25.9 ± 77.7

Treatments were added to THP‐1 cells at a final concentration of 1 μM for 30 min prior to stimulation with 100 pg/mL LPS for 2 h. Data were normalized to GAPDH and compared with VC. Data shown are averages of three independent experiments, each carried out in technical duplicate. Analysis was performed by two‐tailed independent samples *t*‐test.

## 4 Discussion

Flavonoids are perceived to be key components responsible for the observed health effects of fruits and vegetables 30. However, concentrations of individual flavonoid species in the circulation, after “normal/habitual” dietary consumption, are generally extremely low; many orders of magnitude below those observed to elicit cellular activity in in vitro studies 19. We hypothesized that the reported biological activities of flavonoids, following consumption, are not a direct consequence of any individual flavonoid working in isolation, but result from a yet uncharacterized cumulative activity of metabolites, working in combination. We therefore established a screening model to test this hypothesis, examining structurally similar flavonoids and some of their shared metabolites in isolation, and in combinations, as would potentially occur following dietary exposure. Here, we observed some metabolites were more bioactive than their precursor structures and that some combinations of metabolites were more bioactive than their constituent compounds.

The dietary flavonoids utilized in the present investigation are found in abundance in our diets (https://www.gov.uk/government/uploads/system/uploads/attachment_data/file/193804/familyfood‐2011report.pdf). Food sources include onions and tea (quercetin), citrus (naringenin and hesperetin), cocoa/chocolate, red wine and green tea (epicatechin), and red and purple berries (peonidin and cyanidin) 31, 32. Reported levels of consumption vary considerably, and depend on country and population/cohort studied. In cohorts from the United States, Netherlands, and Spain, dietary consumption has been estimated between 158 and 313 mg/day; representing levels for flavan‐3‐ols between 32 and 158 mg/day, flavanones between 14 and 50 mg/day, flavonols between 13 and 21 mg/day, and anthocyanins between 3 and 18 mg/day. However, these estimates represent average consumption, and for many flavonoids considerably higher dietary levels can be achieved from a single serving of a flavonoid‐rich food. For example, anthocyanin concentrations in berries reach 100–700 mg/100 g and it is therefore not unrealistic to consume as much as 250 mg in a single serving 33, 34, 35. Despite potentially high dietary intakes, levels of flavonoids in the blood, postconsumption, are relatively low (generally well below 10 μM 16). Rather than concentrating on dietary flavonoids, the present study focused on the bioactivity of the more physiologically relevant flavonoid metabolites, as phenolic metabolites are reported to be the predominant compounds in the systemic circulation 17, 19, 32, 36. As there is a substantial diversity of phenolic metabolites (or ring‐fission products) reported for the various flavonoids 17, 32, we focused on phenolic acid conjugates common to many flavonoid species, in addition to being found in the habitual diet themselves. These include conjugates of PCA, VA, and BA, which have been reported as phenolic metabolites of berry anthocyanins 17, 19, cocoa flavan‐3‐ols 22, 23, and reported in our recent study of citrus flavanones 37. In addition, phenolic metabolites such as VA and ferulic acid are found in the diet in grain and beer 38, while BA conjugates can be derived from caffeic acid and ferulic acid, which are found in red wine or derived from hydrolysable tannins in the diet 32.

In the current study, we explored bioactivity across a concentration range of 0.1–10 μM, as it represents the relative serum/plasma concentration of flavonoids and their phenolic metabolites reported in the literature. Serum/plasma concentrations of unmetabolized precursor flavonoids are generally quite low, and reported in low to high nanomolar concentrations (10–200 nM) 17, 19, 20, 32, aside from flavanols such as epicatechin, which can reach micromolar concentrations 20. For example, following consumption of 500 mg of the anthocyanin C3G, the phenolic metabolite VA was found to peak at 1.8 μM and persisted at levels of 1 μM for up to 24 h. Similarly, total phenolic metabolites reached cumulative concentrations of 10 μM, in opposition to C3G that reached a peak serum concentration of only 0.1 μM 17, 19. Comparable individual and cumulative plasma concentrations have been reported in studies feeding flavan‐3‐ols, flavanones, and flavonols 22, 23, 39. A 100 μM treatment was also included in the present study to represent a pharmacological dose to reflect maximal responses observed in the literature. Interestingly, doses of 1 μM were consistently the most bioactive of the concentrations tested (Table 2) and when looking across the range of concentrations, U‐shaped trends were often present. These bimodal effects are typical for many phytochemicals 40 and in small dose ranges will appear as either a U‐shaped or inverse U‐shaped dose–response, depending on what part of the curve you capture. The bimodal actions of quercetin have previously been reported in in vitro studies of basophil function 41. In addition, bimodal increases in flow‐mediated dilatation were observed in healthy human volunteers consuming various doses of a beverage rich in blueberries 42, and similar observations were drawn from a recent systematic review of randomized controlled trials involving flavonoid interventions 43.

In the present study, we investigated the effect of the six precursor flavonoids, 14 metabolites, and 29 combinations on LPS‐induced TNF‐α protein levels (Table 2). Initially, we screened 49 treatments across four concentrations in triplicate, representing a total of 588 LPS‐induced TNF‐α protein screens. Two precursor flavonoids (P3G and C3G) and five metabolites (IVA, IVA‐glucuronide, BA‐sulfate, PCA‐3‐sulfate, and VA‐glucuronide) reduced TNF‐α secretion in isolation (Table 2). The screen indicated that the flavonoid metabolites appear more active than their precursor structures, suggesting that the metabolism of flavonoids increases bioactivity in this model.

Four treatments comprising combinations of metabolites (at equal molar ratios) containing: PCA and 4HBA; PCA, 4HBA, and VA; 4HBA, BA‐glucuronide, and BA‐sulfate; and PCA and PCA‐3‐glucuronide significantly reduced TNF‐α secretion at 1–10 μM. Previously combination treatments of chrysin and kaempferol have been reported to be active toward LPS‐stimulated RAW 264.7 cells, reducing nitric oxide, prostaglandin E2, and TNF‐α levels 44, however this previous study focused on the effects of combinations of unmetabolized precursor flavonoids, which is less physiologically relevant, as circulating monocytes are unlikely to be exposed to high concentrations of unmetabolized flavonoids 17, 19, particularly as precursor flavonoids are found at much lower concentrations than their metabolites in the systemic circulation 2, 20.

To allow a direct comparison between the treatments containing combinations of metabolites and their individual constituents, each metabolite was studied individually at the concentration it was present at in the active combination (e.g. 0.5 or 0.33 μM) to establish if there effects were additive. Here PCA, 4HBA, and VA were not active in isolation across the dose ranges explored, suggesting additive effects of the compounds when present together.

Treatments that significantly modulated TNF‐α secretion were further explored for their effect on IL‐1β, as with TNF‐α, IL‐1β is a critical mediator in inflammation where blockade inhibits the progression of cardiovascular disease 8. The treatments that were previously indicated as bioactive in the LPS‐induced TNF‐α screen, had no effect on LPS‐stimulated IL‐1β secretion, with the exception of 4HBA, which significantly reduced IL‐1β secretion by 49.6 ± 12.3% (*p* = 0.011). PCA in isolation also showed a trend toward a reduction in IL‐1β secretion (16.8 ± 15.0%, *p* = 0.171). Studies by Wei et al. and Monagas et al. have previously reported PCA decreasing IL‐1β secretion in LPS‐stimulated murine lung tissue in vivo, and 4HBA reducing IL‐1β secretion in LPS‐stimulated peripheral mononuclear blood cells 45, 46. These results are interesting as both signaling pathways are regulated by NF‐κB, and therefore a similar response would have been anticipated if flavonoids and combinations regulated transcription. In order to further explore the effect of the significant bioactive treatments, TNF‐α and IL‐1β mRNA expression and NF‐κB activation were investigated. There was no effect on mRNA levels, lending credence to the supposition of a post‐transcriptional mode of action. These results are similar to data reported by other groups 47 showing quercetin to have no effect on TNF‐α or IL‐1β mRNA levels in LPS‐stimulated THP‐1 cells. In addition, none of the treatments affected NF‐κB activation, which is similar to previous studies 47, 48, where several flavonoids including naringenin, hesperetin, and epicatechin (0.1–1 μM) and quercetin (33 μM) had no effect on NF‐κB activation in astrocytes or J3774A.1 mouse macrophages, respectively. In contrast, other studies 48, 49 have shown NF‐κB inhibition following treatment with the flavonoids apigenin and luteolin (30 μM) in HEK293 cells and kaempferol‐3‐O‐glucoside (223 μM) in J3774A.1 mouse macrophages; however, none of these flavonoids were investigated in the present study. In addition, the effect on NF‐κB activation was observed at 30–100 μM concentrations of the treatments, which is three‐ to ten‐fold higher than utilized in the present investigation. NF‐κB inhibition may therefore require higher doses of flavonoids, which are likely beyond that of which is physiologically relevant 50 following habitual dietary intake. The effects of the active treatments in the LPS‐induced TNF‐α screen were also explored for their ability to modulate IL‐10 secretion as previously other groups have shown changes in IL‐10 levels 12, 13 following treatment of PMA‐differentiated THP‐1 cells with quince peel extract 12. However, we saw no change in IL‐10 protein secretion compared to the VC. This was also observed in mice fed flavonoid‐rich citrus peel extract for 6 wk 13. IL‐10 is also regulated by NF‐κB.

The protein, mRNA, and reporter assay data all indicate that the flavonoids, metabolites, and combinations act on TNF‐α after transcription. One possible target for future investigation is TNF‐α converting enzyme (TACE). TACE is involved in the post‐translation processing of TNF‐α protein and its activity has been reported to be modulated by the flavonoids rutin, orientin, and isoorietin 51, 52. In contrast, Catalan et al. showed that polyphenol‐rich peanut extract had no significant effect 53; however, future studies should focus on the activity of flavonoid metabolites on TACE. Modulation of TACE would provide an explanation for the different effects between the cytokines, as TACE regulates the post‐translational processing of TNF‐α 54, but has no effect on IL‐1β or IL‐10. IL‐1β 55 is cleaved by caspase 1 to produce its mature soluble form and IL‐10 is not post‐translationally modified 56. Another possible mechanism of action of the flavonoids is inhibition of p38 MAP kinase. p38 MAP kinase regulates TNF‐α protein but not mRNA expression 57 and flavonoids, including procyanidins and epigallocatechin gallate, have previously been reported to inhibit LPS‐induced p38 MAP kinase phosphorylation in macrophages 58, 59, 60, suggesting that this may be a potential mechanism for the inhibitory effects of the flavonoid metabolites on TNF‐α protein but not mRNA expression. These potential mechanisms warrant further investigation.

The present study is not without limitations, most notably, the basic screening model utilized. Cell culture screening methodologies utilizing a pharmacological stimulus such as LPS are most responsive to pharmacologically active substances that have pronounced effects on protein and mRNA expression. Titrating these stimuli to levels, which are more appropriate for studying dietary phytochemical activity, produces less responsive systems, ultimately lowering the dynamic range of the assay, resulting in a larger variability between experiments. Therefore, standard screening models may be less effective in identifying the activity of low concentrations of dietary phytochemicals, which was reflected in the present study by the relatively low responses of the treatments.

The present screen of flavonoid metabolites in isolation and in combination, at low micromolar concentrations, is a novel design and provides an important contribution to the field, as it aims to explore the activity of a group of physiologically relevant phytochemical metabolites at appropriate concentrations. The study of complex mixtures of metabolites, in addition to utilizing pure synthesized metabolites is also quite unique. However, as there is a substantially high day‐to‐day variation in dietary flavonoid consumption between individuals, and enormous interindividual variation reported for the absorption, distribution, metabolism, and elimination of flavonoids 17, 19, designing a treatment that entirely mimics plasma composition is unrealistic. Because of this actuality, we selected a more practical approach of exploring the activity of a number of simple and complex mixtures of structurally similar flavonoids and metabolites across a concentration range, in order to gain insight into structure–activity relationships and additive and/or synergistic activities. As the concentrations of each individual analyte would differ with diet and individual, it was also practical to use equal concentrations of the analytes in the mixtures. Most in vitro studies in the past have investigated the actions of supraphysiological concentrations of precursor flavonoids in isolation, or using crude fruit or serum extracts. Here, our screen has revealed possible modes of action of flavonoid metabolites, however, further work is needed to fully ascertain the mechanisms of action. Interestingly, the greatest activities observed appeared to be at the lowest concentrations investigated and appeared nonlinear, which could suggest high‐dose interventions may not be necessary for prevention or disease risk‐reduction. Ultimately, the present study suggests that the health benefits observed in epidemiological studies, which have been accredited to flavonoid consumption, may be the result of the activity of complex combinations of flavonoids and their metabolites, as found in the systemic circulation following normal dietary consumption.

In conclusion, we have shown that some metabolites of flavonoids individually and in combination appear more bioactive then their precursor flavonoids, at equivalent concentrations. In time, these data may help establish appropriate combinations of flavonoid‐rich foods for utilization in animal and human trials exploring dietary strategies for cardio‐protection.

## Supporting information

As a service to our authors and readers, this journal provides supporting information supplied by the authors. Such materials are peer reviewed and may be re‐organized for online delivery, but are not copy‐edited or typeset. Technical support issues arising from supporting information (other than missing files) should be addressed to the authors.

**Table S1** Effect of treatments on LPS‐induced TNF‐α protein secretion with including all data and p values.
**Table S2**. Effect of treatments on IL‐1β protein expression including p values.
