# Supplementary material for: Flavonoid metabolites reduce tumor necrosis factor‐α secretion to a greater extent than their precursor compounds in human THP‐1 monocytes
Source: Mol Nutr Food Res. 2015 May 3;59(6):1143–54. doi: 10.1002/mnfr.201400799 (PMC4973837; doi:10.1002/mnfr.201400799)
Supplement: Supplementary file 1 — Table S1 Effect of treatments on LPS‐induced TNF‐α protein secretion with including all data and p values. Table S2. Effect of treatments on IL‐1β protein expression including p values. [file MNFR-59-1143-s001.docx]

Supplemental Data

Table S1 Effect of treatments on LPS-induced TNF-α protein secretion with including all data and p values.

| Treatment | Average change in TNF-α protein from VC (% ± SD) | | | | | |
| --- | --- | --- | --- | --- | --- | --- |
|  | 0.1 µM | P value | 1 µM | P value | 10 µM | P value |
| Naringenin | ↓ 17.0 ± 7.2 | 0.358 | ↓ 24.2 ± 3.3 | 0.199 | ↓ 29.0 ± 18.3 | 0.131 |
| Quercetin | ↓ 24.9 ± 7.1 | 0.375 | ↓27.0 ± 19.0 | 0.338 | ↓ 19.8 ± 8.7 | 0.478 |
| Epicatechin | ↓ 19.1 ± 10.7 | 0.174 | ↓ 25.7 ± 13.1 | 0.077 | ↓ 28.5 ± 31.0 | 0.054 |
| C3G | ↓ 9.5 ± 2.4 | 0.147 | **↓ 13.7 ± 4.4*** | **0.047** | ↓ 12.2 ± 1.2 | 0.071 |
| Hesperetin | ↓ 18.0 ± 4.4 | 0.414 | ↓ 23.2 ± 15.0 | 0.300 | ↓ 26.2 ± 8.2 | 0.245 |
| P3G | **↓ 12.7 ± 4.2*** | **0.017** | **↓ 19.8 ± 3.1***** | **≤0.001** | **↓ 13.4 ± 6.5*** | **0.013** |
| 4HBA | ↓ 2.3 ± 12.8 | 0.869 | ↓ 10.9 ± 12.9 | 0.446 | ↓ 8.4 ± 11.6 | 0.556 |
| PCA | ↓ 9.6 ± 20.8 | 0.538 | ↓ 24.3 ± 13.9 | 0.139 | ↓ 20.5 ± 36.3 | 0.205 |
| IVA | ↓ 12.8 ± 3.3 | 0.248 | **↓ 27.0 ± 3.5*** | **0.027** | ↓ 20.2 ± 5.7 | 0.083 |
| VA | ↓ 0.1 ± 33.8 | 0.991 | ↓ 13.5 ± 30.7 | 0.308 | ↓ 12.6 ± 29.9 | 0.337 |
| BA-glucuronide | ↓ 8.2 ± 3.8 | 0.541 | ↓ 11.0 ± 4.4 | 0.415 | ↓ 16.5 ± 6.8 | 0.232 |
| BA-sulfate | ↓ 24.9 ± 32.5 | 0.147 | **↓ 37.2 ± 40.1*** | **0.041** | ↓ 27.3 ± 38.5 | 0.117 |
| PCA-3-glucuronide | ↓ 1.8 ± 15.0 | 0.894 | ↓ 18.9 ± 5.6 | 0.187 | ↓ 15.6 ± 4.5 | 0.270 |
| PCA-4-glucuronide | ↓ 7.1 ± 3.8 | 0.722 | ↓ 5.8 ± 18.5 | 0.771 | ↓ 12.0 ± 6.9 | 0.549 |
| PCA-3-sulfate | ↓ 24.6 ± 33.8 | 0.123 | **↓ 41.1 ± 42.0*** | **0.018** | **↓ 38.6 ± 43.0*** | **0.025** |
| PCA-4-sulfate | ↑ 9.1 ± 14.3 | 0.558 | ↓ 6.1 ± 10.3 | 0.695 | ↑ 0.1 ± 11.1 | 0.995 |
| IVA-glucuronide | **↓ 16.8 ± 15.1*** | **0.037** | **↓ 32.8 ± 9.8***** | **≤0.001** | **↓ 33.3 ± 11.8***** | **≤0.001** |
| IVA-sulfate | ↓ 0.6 ± 15.5 | 0.972 | ↓ 17.5 ± 11.0 | 0.317 | ↓ 8.2 ± 8.0 | 0.634 |
| VA-glucuronide | ↓ 12.5 ± 15.7 | 0.251 | **↓ 26.9 ± 19.4*** | **0.025** | **↓ 26.8 ± 4.9*** | **0.026** |
| VA-sulfate | ↓ 1.8 ± 9.0 | 0.919 | ↓ 20.0 ± 8.0 | 0.270 | ↓ 12.1 ± 6.4 | 0.494 |
| Combination 1 | ↓ 17.1 ± 13.1 | 0.332 | ↑ 23.1 ± 2.3 | 0.198 | ↓ 6.0 ± 13.4 | 0.729 |
| Combination 2 | ↑ 28.4 ± 39.3 | 0.225 | ↓ 12.4 ± 16.5 | 0.584 | ↓ 18.1 ± 16.3 | 0.430 |
| Combination 3 | ↓ 66.6 ± 87.0 | 0.096 | ↓ 28.8 ± 39.2 | 0.445 | ↓ 14.6 ± 11.5 | 0.696 |
| Combination 4 | ↓ 48.3 ± 89.0 | 0.201 | ↓ 71.6 ± 75.8 | 0.070 | ↓ 6.9 ± 19.5 | 0.849 |
| Combination 5 | ↓ 27.5 ± 28.7 | 0.731 | ↓ 38.8 ± 73.5 | 0.629 | ↓59.8 ± 142.4 | 0.461 |
| Combination 6 | ↓ 18.3 ± 21.2 | 0.824 | ↓ 74.0 ± 118.6 | 0.379 | ↓79.7 ± 125.6 | 0.345 |
| Combination 7 | ↓12.6 ± 28.2 | 0.659 | ↓ 9.9 ± 26.0 | 0.729 | ↓ 31.3 ± 19.9 | 0.286 |
| Combination 8 | ↓ 12.2 ± 10.6 | 0.322 | ↓ 20.8 ± 13.9 | 0.107 | ↓ 11.1 ± 3.5 | 0.368 |
| Combination 9 | **↓ 26.3 ± 6.4*** | **0.019** | **↓ 33.1 ± 6.1**** | **0.006** | **↓ 42.7 ± 13.5***** | **≤0.001** |
| Combination 10 | ↓ 17.9 ± 9.8 | 0.135 | **↓ 47.4 ± 12.3**** | **0.002** | **↓35.1 ± 5.1**** | **0.010** |
| Combination 11 | ↓ 2.0 ± 13.1 | 0.911 | ↓ 15.9 ± 6.8 | 0.377 | ↓ 8.9 ± 25.1 | 0.616 |
| Combination 12 | ↑ 16.7 ± 35.1 | 0.526 | ↓ 3.3 ± 18.0 | 0.898 | ↓ 9.4 ± 23.0 | 0.720 |
| Combination 13 | ↓ 10.3 ± 29.0 | 0.475 | ↓ 26.2 ± 37.6 | 0.088 | **↓ 32.0 ± 35.4*** | **0.043** |
| Combination 14 | ↓ 2.0 ± 16.9 | 0.503 | ↓ 1.9 ± 32.3 | 0.214 | ↓ 3.0 ± 8.8 | 0.686 |
| Combination 15 | ↓ 11.6 ± 9.0 | 0.969 | ↓ 22.2 ± 13.7 | 0.109 | ↓ 7.0 ± 24.6 | 0.061 |
| Combination 16 | ↓ 1.1 ± 19.0 | 0.124 | **↓ 6.5 ± 21.1*** | **0.049** | **↓ 4.4 ± 13.4*** | **0.032** |
| Combination 17 | ↓ 0.7 ± 50.5 | 0.972 | ↓ 30.3 ± 40.8 | 0.840 | ↓ 36.4 ± 34.2 | 0.450 |
| Combination 18 | ↓ 29.5 ± 34.9 | 0.936 | ↓ 39.3 ± 26.4 | 0.308 | ↓ 43.8 ± 39.9 | 0.991 |
| Combination 19 | ↓ 1.0 ± 61.0 | 0.726 | ↓ 6.0 ± 60.1 | 0.790 | ↓ 22.6 ± 61.8 | 0.806 |
| Combination 20 | ↓ 1.1 ± 0.9 | 0.682 | ↓ 14.2 ± 8.6 | 0.468 | ↑ 0.2 ± 16.3 | 0.380 |
| Combination 21 | ↑ 6.3 ± 6.6 | 0.630 | ↑ 4.8 ± 10.1 | 0.655 | ↑ 4.4 ± 9.6 | 0.539 |
| Combination 22 | ↓ 4.7 ± 10.6 | 0.687 | ↓ 8.5 ± 11.9 | 0.392 | ↓ 10.3 ± 2.6 | 0.118 |
| Combination 23 | ↑ 8.4 ± 15.1 | 0.778 | ↓ 7.8 ± 19.2 | 0.914 | ↓ 10.8 ± 17.2 | 0.869 |
| Combination 24 | ↑ 4.5 ± 9.6 | 0.703 | ↓ 9.7 ± 18.2 | 0.267 | ↓ 18.5 ± 9.9 | 0.345 |
| Combination 25 | ↑ 3.7 ± 24.5 | 0.948 | ↑ 1.4 ± 24.6 | 0.400 | ↑ 2.2 ± 35.5 | 0.458 |
| Combination 26 | ↑ 13.5 ± 64.8 | 0.727 | ↓ 40.2 ± 44.0 | 0.435 | ↓ 33.9 ± 24.5 | 0.749 |
| Combination 27 | ↓ 1.8 ± 64.4 | 0.622 | ↓ 23.1 ± 40.7 | 0.875 | ↓ 20.3 ± 37.0 | 0.620 |
| Combination 28 | ↑ 15.6 ± 53.0 | 0.900 | ↑35.4 ± 85.5 | 0.904 | ↓14.3 ± 8.1 | 0.852 |
| Combination 29 | ↑ 19.0 ± 42.0 | 0.934 | ↑ 6.0 ± 35.8 | 0.419 | ↑ 19.1 ± 44.3 | 0.747 |

Treatments (see Table 1 for details) were added to THP-1 cells at final concentrations of 0.1, 1 and 10 µM for 30 min prior to stimulation with 100 pg/mL LPS for 3 h LPS-induced TNF-α protein secretion was quantified by ELISA. Data shown are averages of 3 independent experiments, each carried out in technical duplicate. Analysis was performed by one-way ANOVA and post-hoc LSD, *p <0.05, **p <0.01, ***p≤0.001.

Table S2. Effect of treatments on IL-1β protein expression including p values.

| Treatment | Average change in IL-1β protein from vehicle control (% ± SD) | |
| --- | --- | --- |
|  | 1 µM | P value |
| C3G | ↓ 1.3 ± 28.9 | 0.967 |
| P3G | ↓ 15.2 ± 20.6 | 0.502 |
| IVA | ↑ 19.2 ± 10.2 | 0.281 |
| IVA-glucuronide | ↑ 24.3 ± 15.3 | 0.391 |
| VA-glucuronide | ↓ 1.7 ± 50.3 | 0.967 |
| PCA-3-sulfate | ↓ 31.2 ± 3.6 | 0.087 |
| BA-sulfate | ↓ 28.9 ± 35.0 | 0.289 |
| Combo 9 | ↓ 8.7 ± 17.8 | 0.811 |
| Combo 10 | ↑ 15.5 ± 30.9 | 0.682 |
| Combo 13 | ↓ 6.0 ± 11.7 | 0.595 |
| Combo 16 | ↓ 10.3 ± 22.3 | 0.571 |
| VA | ↓ 23.0 ± 44.4 | 0.335 |
| PCA | ↓ 16.8 ± 15.0 | 0.171 |
| 4HBA | **↓ 49.6 ± 12.3*** | **0.011*** |
| BA-glucuronide | ↓ 2.6 ± 10.5 | 0.765 |
| PCA-3-glucuronide | ↓ 2.0 ± 22.1 | 0.956 |

Treatments were added to THP-1 cells at a final concentration of 1 µM for 30 min prior to stimulation with 10 µg/mL LPS for 24 h. Data were normalised to LPS and compared with VC. Data shown are averages of 3 independent experiments, each carried out in technical duplicate. Analysis was performed by two-tailed independent samples t-test *p<0.05.

Table S3 Effect of treatments on TNF-α mRNA expression including p values.

| Treatment | Average change in TNF-α mRNA from vehicle control (% ± SD) | |
| --- | --- | --- |
|  | 1 µM | P value |
| C3G | ↑ 0.8 ± 13.0 | 0.914 |
| P3G | ↓ 7.0 ± 28.7 | 0.614 |
| IVA | ↓ 2.8 ± 62.7 | 0.940 |
| IVA-glucuronide | ↑ 17.1 ± 51.8 | 0.593 |
| VA-glucuronide | ↑ 6.3 ± 27.1 | 0.650 |
| PCA-3-sulfate | ↓ 1.3 ± 21.7 | 0.911 |
| BA-sulfate | ↓ 8.8 ± 33.2 | 0.605 |
| Combo 9 | ↑ 17.7 ± 36.0 | 0.715 |
| Combo 10 | ↑ 9.5 ± 28.7 | 0.789 |
| Combo 13 | ↓ 35.9 ± 65.8 | 0.428 |
| Combo 16 | ↑ 3.6 ± 56.1 | 0.912 |
| VA | ↑ 20.2 ± 55.5 | 0.717 |
| PCA | ↑ 17.5 ± 36.0 | 0.635 |
| 4HBA | ↑ 44.5 ± 64.6 | 0.508 |
| BA-glucuronide | **↑ 23.8 ± 6.5** | **0.050*** |
| PCA-3-glucuronide | ↑ 25.9 ± 77.7 | 0.723 |

Treatments were added to THP-1 cells at a final concentration of 1 µM for 30 min prior to stimulation with 100 pg/mL LPS for 2 h. Data were normalised to GAPDH and compared with VC. Data shown are averages of 3 independent experiments, each carried out in technical duplicate. Analysis was performed by two-tailed independent samples t-test.
